# Effects of 25-Hydroxyvitamin D_3_ on Proliferation and Osteoblast Differentiation of Human Marrow Stromal Cells Require CYP27B1/1α-Hydroxylase

**DOI:** 10.1002/jbmr.298

**Published:** 2010-11-23

**Authors:** Shuo Geng, Shuanhu Zhou, Julie Glowacki

**Affiliations:** 1 Department of Orthopedic Surgery, Brigham and Women's Hospital, Harvard Medical SchoolBoston, MA, USA; 2 Department of Orthopedic Surgery, First Affiliated Hospital of Harbin Medical University, Harbin Medical UniversityHarbin, Heilongjiang, People's Republic of China

**Keywords:** BONE MARROW STROMAL CELLS, VITAMIN D, PROLIFERATION, OSTEOBLAST DIFFERENTIATION, APOPTOSIS

## Abstract

1,25-Dihydroxyvitamin D_3_ [1,25(OH)_2_D_3_] has many noncalcemic actions that rest on inhibition of proliferation and promotion of differentiation in malignant and normal cell types. 1,25(OH)_2_D_3_ stimulates osteoblast differentiation of human marrow stromal cells (hMSCs), but little is known about the effects of 25-hydroxyvitamin D_3_ [25(OH)D_3_] on these cells. Recent evidence shows that hMSCs participate in vitamin D metabolism and can activate 25(OH)D_3_ by CYP27B1/1α-hydroxylase. These studies test the hypothesis that antiproliferative and prodifferentiation effects of 25(OH)D_3_ in hMSCs depend on CYP27B1. We studied hMSCs that constitutively express high (hMSCs^hi-1α^) or low (hMSCs^lo-1α^) levels of CYP27B1 with equivalent expression of CYP24A1 and vitamin D receptor. In hMSCs^hi-1α^, 25(OH)D_3_ reduced proliferation, downregulated proliferating cell nuclear antigen (PCNA), upregulated p21^Waf1/Cip1^, and decreased cyclin D1. Unlike 1,25(OH)_2_D_3_, the antiapoptotic effects of 25(OH)D_3_ on Bax and Bcl-2 were blocked by the P450 inhibitor ketoconazole. The antiproliferative effects of 25(OH)D_3_ in hMSCs^hi-1α^ and of 1,25(OH)_2_D_3_ in both samples of hMSCs were explained by cell cycle arrest, not by increased apoptosis. Stimulation of osteoblast differentiation in hMSCs^hi-1α^ by 25(OH)D_3_ was prevented by ketoconazole and upon transfection with *CYP27B1* siRNA. These data indicate that CYP27B1 is required for 25(OH)D_3_'s action in hMSCs. Three lines of evidence indicate that CYP27B1 is required for the antiproliferative and prodifferentiation effects of 25(OH)D_3_ on hMSCs: Those effects were not seen (1) in hMSCs with low constitutive expression of *CYP27B1*, (2) in hMSCs treated with ketoconazole, and (3) in hMSCs in which *CYP27B1* expression was silenced. Osteoblast differentiation and skeletal homeostasis may be regulated by autocrine/paracrine actions of 25(OH)D_3_ in hMSCs. © 2011 American Society for Bone and Mineral Research.

## Introduction

Vitamin D is an important regulator of mineral and bone metabolism, and it is now appreciated that its metabolites and analogues have many other actions. Calcitriol, or 1α,25-dihydroxyvitamin D_3_ [1,25(OH)_2_D_3_], is the most active metabolite, with high affinity for the nuclear vitamin D receptor (VDR).(1) It is produced in the kidney by the 1α-hydroxylation of the precursor 25-hydroxyvitamin D_3_ [25(OH)D_3_] by the cytochrome P450 enzyme CYP27B1/1α-hydroxylase.(2) Hydroxylation of vitamin D metabolites at the carbon 24 position by 25-hydroxyvitamin D–24-hydroxylase (CYP24A1) is the first step in their inactivation and excretion. Basal expression of CYP24A1 is usually low but is highly induced by 1,25(OH)_2_D_3_.(1)

Calcitriol has major effects in inhibiting proliferation and promoting differentiation of many cell types, especially tumor cells such as human breast cancer cells,(3) colon sarcoma cells,(4) prostate cancer cells, colorectal adenoma, and carcinoma cells.(5) Epidemiologic and experimental studies also indicate that 1,25(OH)_2_D_3_ has antitumor effects(6); those effects are attributed to the inhibition of proliferation,(5,7,8) arrest of cell cycle,(3,9) increase in apoptosis,(4,10,11) and induction of differentiation.(12,13) The antiproliferative and prodifferentiation effects of 1,25(OH)_2_D_3_ also have been demonstrated for some nonmalignant cell types, such as human peripheral monocytes.(14,15) Little is known, however, about the effects of 25(OH)D_3_ on cell proliferation and differentiation.

In addition to kidney tubule cells, other human cells, notably osteoblasts(16) and their progenitors in the bone marrow,(17) produce 1,25(OH)_2_D_3_. Bone cells participate in vitamin D metabolism and also are targets of 1,25(OH)_2_D_3_ action.(17) The differentiation of human marrow stromal cells (hMSCs, aka mesenchymal stem cells)(18) and rat osteogenic ROS 17/2 cells(19) to osteoblasts is stimulated by 1,25(OH)_2_D_3_. Less is known about the effects of 25(OH)D_3_ on bone cells. In recent studies with freshly isolated hMSCs from 19 subjects, 1,25(OH)_2_D_3_ stimulated osteoblast differentiation in all samples, and 25(OH)D_3_ did so in two-thirds of them.(17) The variability in response to 25(OH)D_3_ may be due to differences in expression of CYP27B1. The combined presence of CYP27B1 and VDR indicates possible autocrine/paracrine roles for 25(OH)D_3_ in hMSCs. This study tests the hypothesis that the antiproliferative and prodifferentiation effects of 25(OH)D_3_ in hMSCs depend on CYP27B1.

## Materials and Methods

### Cells and reagents

Bone marrow samples were obtained with institutional review board approval as femoral tissue discarded during primary hip arthroplasty for osteoarthritis. A series of samples from 22 subjects (average age is 58 ± 15 years) was prepared and screened. Low-density marrow mononuclear cells were isolated by centrifugation on Ficoll/Histopaque 1077 (Sigma, St Louis, MO, USA).(20) This procedure removes differentiated cells and enriches for undifferentiated low-density marrow mononuclear cells that include a population of nonadherent hematopoietic cells and a fraction capable of adherence and differentiation into musculoskeletal cells. The nonadherent hematopoietic stem cells were rinsed away 24 hours after seeding, and the adherent hMSCs were expanded in monolayer culture with standard growth medium, phenol red–free α modified essential medium α-MEM), 10% fetal bovine serum–heat inactivated (FBS-HI), 100 U/mL of penicillin, and 100 µg/mL of streptomycin (Invitrogen, Carlsbad, CA, USA). All samples were used at passages 2 through 4. Some experiments used standard osteogenic medium (ie, phenol red–free α-MEM, 10% FBS-HI, 100 U/mL of penicillin, 100 µg/mL of streptomycin, 10 nM dexamethasone, 5 mM β-glycerophosphate, and 50 µg/mL of ascorbate-2-phosphate) or osteogenic medium (ie, phenol red–free α-MEM, 1% FBS-HI, 100 U/mL of penicillin, 100 µg/mL of streptomycin, 10 nM dexamethasone, 5 mM β-glycerophosphate, and 50 µg/mL of ascorbate-2-phosphate). After transfection with siRNA, all media used were without 100 U/mL of penicillin and 100 µg/mL of streptomycin. Reagents such as 25(OH)D_3_, 1,25(OH)_2_D_3_, and ketoconazole were purchased from Sigma; each was prepared as a stock solution at 10^−3^ M in absolute ethanol and stored at −80°C. In preliminary dose-finding studies (data not shown) with Western immunoblotting, we found no responses to 1, 10, or 100 nM 25(OH)D_3_ and responses to 1000 nM 25(OH)D_3_; thus most experiments used 1000 nM 25(OH)D_3_. In all 3-day experiments, vitamin D metabolites were added daily to control for inactivation by 24-hydroxylation.

### RNA isolation and RT-PCR

Total RNA was isolated from human MSCs with TRIZOL reagent (Invitrogen). For reverse-transcriptase polymerase chain reaction (RT-PCR), 2 µg of total RNA was reverse-transcribed into cDNA with SuperScript II (Invitrogen) following the manufacturer's instructions. Concentrations of cDNA and amplification conditions were optimized for each gene product to reflect the exponential phase of amplification. One-twentieth of the cDNA was used in each 50-µL PCR reaction (30 to 40 cycles of 94°C for 1 minute, 55 to 60°C for 1 minute, and 72°C for 2 minutes), as described previously.(20) Gene-specific primer pairs (Table 1) for *CYP27B1*,(17) *CYP24A1*,(17) *VDR*,(17) *Cbfa1/Runx2* (*Runx2*),(21) *AlkP*,(21) *bone sialoprotein* (*BSP*),(21) *Bax*,(21) and *Bcl*-2(22) were used for amplification. PCR products were separated by agarose gel electrophoresis and were quantified by densitometry of captured gel images with a Kodak Gel Logic 200 Imaging System and Kodak Molecular Imaging Software following the manufacturer's instructions (Kodak Molecular Imaging Systems, Rochester, NY, USA). Data were expressed by normalizing the densitometric units to *GAPDH* (internal control).

**Table 1:** Primer Sets Used for RT-PCR

Accession number	Primer name	Sequence (5′→3′)	Product size (bp)
NM_000785.3	*CYP27B1*	F = GCTACACGAGCTGCAGGTGCAGGGC	252
		R = AGCGGGGCCAGGAGACTGCGGAGCC	
NM_001128915.1	*CYP24A1*	F = GCAGCCTAGTGCAGATTT	335
		R = ATTCACCCAGAACTGTTG	
NM_001017535.1	*VDR*	F = AGCCTCAATGAGGAG CACTCCAAG	208
		R = ACGGGTGAGGAGGGCTGCTGAGTA	
NM_004324.3	*Bax*	F = GAGGATGATTGCCGCCGTGGAC	279
		R = CGGTGGTGGGGGTGAGGAGG	
NM_000633.2	*Bcl-*2	F = CTTTCCATGTTGTTGGCCGGATCA	137
		R = CCCAGGGCAAAGAAATGCAAGTGA	
NM_001015051.3	*Runx2*	F = GTTTGTTCTCTGACCGCCTC	318
		R = CCAGTTCTGAGGCACCTGAAA	
NM_000478.4	*AlkP*	F = GCGAACGTATTTCTCCAGACCCAG	369
		R = TTCCAAACAGGAGAGTCGCTTCA	
NM_004967	*BSP*	F = TCAGCATTTTGGGAATGGCC	657
		R = GAGGTTGTTGTCTTCGAGGT	
NM_002046.3	*GAPDH*	F = TGATGACATCAAGAAGGTGGTGAAG	240
		R = TCCTTGGAGGCCATGTGGGCCAT	

### In vitro biosynthesis of 1,25(OH)_2_D_3_ by hMSCs

For comparing synthesis of 1,25(OH)_2_D_3_, hMSCs (three replicate wells) were cultivated in 12-well plates until confluence, and then the medium was changed to serum-free α-MEM supplemented with 1% insulin-transferrin-selenium plus linoleic-bovine serum albumin (ITS)^+1^, 10 µM 1,2-dianilinoethane (*N*,*N*'-diphenylethylene diamine; Sigma) and treated with or without 1000 nM 25(OH)D_3_ for 24 hours.(17) This concentration of substrate 25(OH)D_3_ is customary for in vitro biosynthesis studies.(17,23) 1,2-Dianilinoethane was added to the cultures as an antioxidant. Supernatants were harvested and stored at −20°C prior to analysis for 1,25(OH)_2_D_3_ content. The 1,25(OH)_2_D_3_ levels in the media were determined quantitatively with a 1,25(OH)_2_D_3_ EIA kit (Immunodiagnostic Systems, Ltd., Fountain Hills, AZ, USA) according to the manufacturer's instructions. The hMSCs were lysed with a buffer containing 150 mM NaCl, 3 mM NaHCO_3_, 0.1% Triton X-100, and a mixture of protease inhibitors (Roche Diagnostics, Mannheim, Germany). Protein concentration was determined with the BCA System (Thermo Fisher Scientific, Rockford, IL, USA). The CYP27B1 activity was expressed as biosynthesized 1,25(OH)_2_D_3_ in medium per milligram of protein per hour (femtomoles per milligram of protein per hour).

### Proliferation

Human MSCs (hMSCs^hi-1α^ and hMSCs^lo-1α^) at passage 2 were seeded at 3000/cm^2^ in 12-well plates. Cells were cultured in replicate (12 replicate wells) in standard growth medium (10% FBS-HI) in the absence or presence of 1, 10, or 100 nM 1,25(OH)_2_D_3_ or 25(OH)D_3_ for 3 days. Cells were suspended with 0.5 mL of 0.05% trypsin-ethylenediamine tetraacetic acid (Invitrogen), and cell number was determined by hemacytometer.

### Western immunoblot

Human MSCs were cultured in 100-mm dishes in standard growth medium (10% FBS-HI). At 50% confluence, the cells were treated with 1, 10, 100 nM 1,25(OH)_2_D_3_ or 1000 nM 25(OH)D_3_ for 3 days. Whole-cell lysates were prepared with lysis buffer (150 mM NaCl, 3 mM NaHCO_3_, 0.1% Triton X-100, and a mixture of protease inhibitors; Roche Diagnostics, Mannheim, Germany) and were homogenized with a pestle (Kontes, Vineland, NJ, USA) and centrifuged at 16,000*g* (Eppendorf centrifuge; Eppendorf, Hamburg, Germany). Protein concentration was determined (BCA system; Thermo Fisher Scientific). Western immunoblotting was performed as described previously.(20) In brief, proteins were resolved on 4% to 12% SDS-PAGE (NuPAGE Bis-Tris gel; Invitrogen) and transferred onto polyvinylidene fluoride membranes (PVDF; Amersham Biosciences, Piscataway, NJ, USA). The membranes were blocked with 5% nonfat milk in PBS buffer containing 0.1% Tween-20 (PBST) for 2 to 3 hours at room temperature and incubated at 4°C overnight with primary antibodies proliferating cell nuclear antigen (PCNA) (1:3000; Abcam, Cambridge, UK), CYP27B1 (H-90, 1:1000; Santa Cruz Biotechnology, Inc., Santa Cruz, CA, USA), β-actin (1:8000, Santa Cruz Biotechnology, Inc.), and Bax, Bcl-2, p21^Waf1/Cip1^, and cyclin D1 (each at 1:1000; Cell Signaling Technology, Beverly, MA, USA). After removal of the unbound primary antibodies by three 5-minute washes with PBST, the membranes were incubated with horseradish peroxidase–conjugated secondary antibodies (1:5000) for 1 hour at room temperature and washed three times for 5 minutes with PBST. The antibody-associated protein bands were revealed with the ECL-plus Western blotting system (Amersham Biosciences).

### Alkaline phosphatase (AlkP) enzymatic activity assay

For AlkP enzymatic activity assay, the concentration of serum in standard osteogenic medium (10% FBS-HI) was reduced to 1% FBS-HI to minimize possible subsequent differences in proliferation that could confound interpretation of the effects of vitamin D metabolites on osteoblastogenesis. The medium was changed every 2 days. AlkP enzyme activity was measured spectrophotometrically, as described previously.(21) Protein concentration was determined with the BCA system (Thermo Fisher Scientific, Inc.). The AlkP enzyme activity was expressed as micromoles per minute per gram of protein, and some was calculated as the ratio of treated relative to control.

### RNA interference with *CYP27B1* siRNA

Transient transfection of siRNA into hMSCs^hi-1α^ was performed by electroporation with the Human MSC Nucleofector Kit (Lonza/Amaxa Biosystems, Walkersville, MD, USA) with either *CYP27B1* siRNA, nonsilencing control siRNA (a nonhomologous, scrambled sequence equivalent; Santa Cruz Biotechnology, Inc.), or PBS according to the manufacturer's instructions and as described previously.(24) In brief, hMSCs^hi-1α^ were harvested by trypsinization and resuspended at 10^6^ cells in 100 µL of Nucleofector Solution (Lonza/Amaxa Biosystems) with 10 or 100 pmol of *CYP27B1* siRNA. Electroporation was performed in Nucleofector II device with Program U-23 (Lonza/Amaxa Biosystems). Immediately after electroporation, the cells were transferred to 60-mm dishes or 12-well plates in phenol red–free α-MEM and 10% FBS-HI. Some cells were collected at 80% confluence for RT-PCR or Western immunoblot analysis to determine the effect of *CYP27B1* siRNA. Some cells that were cultured until confluent in the 12-well plates were treated with or without 1000 nM 25(OH)D_3_ in serum-free α-MEM supplemented with 1% ITS^+1^, 10 µM 1,2-dianilinoethane (*N*,*N*'-diphenylethylene diamine) for 24 hours to assess 1α-hydroxlyase activity. Cellular 1,25(OH)_2_D_3_ production was determined by EIA as described under “In vitro biosynthesis of 1,25(OH)_2_D_3_ by hMSCs.” At 24 hours after electroporation, some cells were treated with either 25(OH)D_3_ (1000 nM) or vehicle control (ethanol) daily in standard growth medium (10% FBS-HI) for another 72 hours for RT-PCR assays. When some cells in the 12-well plates were nearly 80% confluent, the medium was changed to the osteogenic medium with 1% FBS-HI ± 10 nM 25(OH)D_3_ for 7 days for assessment of alkP enzymatic activity as another index of osteoblast differentiation.

### Statistical analysis

Experiments were performed at least in triplicate. Group data are presented as mean ± SEM unless otherwise indicated. Quantitative data were analyzed with nonparametric tools, either the Mann-Whitney test or Spearman correlation test. If data allowed, parametric tools were used, either *t* test for two group or one-way ANOVA for multiple group comparisons or Pearson correlation test. A value of *p* < .05 was considered significant.

## Results

### Expression of *CYP27B1* and *CYP24A1* genes and 1α-hydroxylase activity in hMSCs

**Fig. 1:**
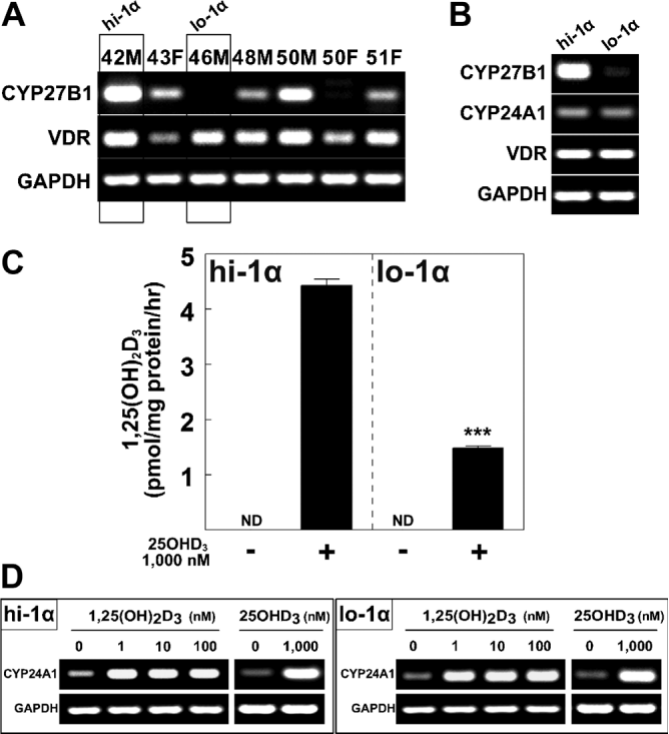
Expression of *CYP27B1* and *CYP24A1* genes and 1α-hydroxylase activity in hMSCs. (*A*) Gel electrophoretogram shows RT-PCR products *CYP27B1*, *VDR*, and *GAPDH* in 7 representative specimens of hMSCs. Labels for lanes indicate age and gender. (*B*) Gel electrophoretogram shows RT-PCR products for *CYP27B1*, *CYP24A1*, *VDR*, and *GAPDH* in selected hMSCs^hi-1α^ (from a 42-year-old man) and hMSCs^lo-1α^ (from a 46-year-old man). (*C*) 1,25(OH)_2_D_3_ synthesis was measured in hMSCs^hi-1α^ and hMSCs^lo-1α^. Cultures were treated with or without 1000 nM 25(OH)D_3_ in serum-free α-MEM supplemented with 1% ITS^+1^, 10 µM 1,2-dianilinoethane (*N*,*N*'-diphenylethylene diamine) for 24 hours. Cellular 1,25(OH)_2_D_3_ production (three replicate wells) was determined by EIA. There was no detectable (ND) 1,25(OH)_2_D_3_ in cultures without 25(OH)D_3_ exogenous substrate. ***p < .001. (*D*) Gel electrophoretogram shows RT-PCR products for *CYP24A1* and *GAPDH* in hMSC^hi-1α^ and hMSC^lo-1α^ cultures after 3 days in standard growth medium with 10% FBS-HI in the absence or presence of 1,25(OH)_2_D_3_ or 25(OH)D_3_.

Gene expression analysis with hMSCs from 22 subjects showed a wide range of constitutive expression of CYP27B1 (Fig. 1*A*, showing 7 representative samples). Two samples of hMSCs were selected for detailed studies, having either high (hMSCs^hi-1α^, from a 42-year-old man) or low (hMSCs^lo-1α^, from a 46-year-old man) levels of CYP27B1, with equivalent expression of CYP24A1 and VDR (Fig. 1*B*). Their activity for 1α-hydroxylation was compared by measuring production of 1,25(OH)_2_D_3_. Biosynthesis of 1,25(OH)_2_D_3_ in the hMSCs^hi-1α^ was 2.98-fold greater that in the hMSCs^lo-1α^ (4433.4 versus 1487.9 fmol/mg protein per hour, *p* < .0001; Fig. 1*C*). Upregulation of CYP24A1 by 1,25(OH)_2_D_3_ (1, 10, and 100 nM) or 25(OH)D_3_ (1000 nM) treatment was equivalent in both specimens of hMSCs (Fig. 1*D*).

### Relative antiproliferative effects of 25(OH)D_3_ and 1,25(OH)_2_D_3_ on hMSCs

**Fig. 2:**
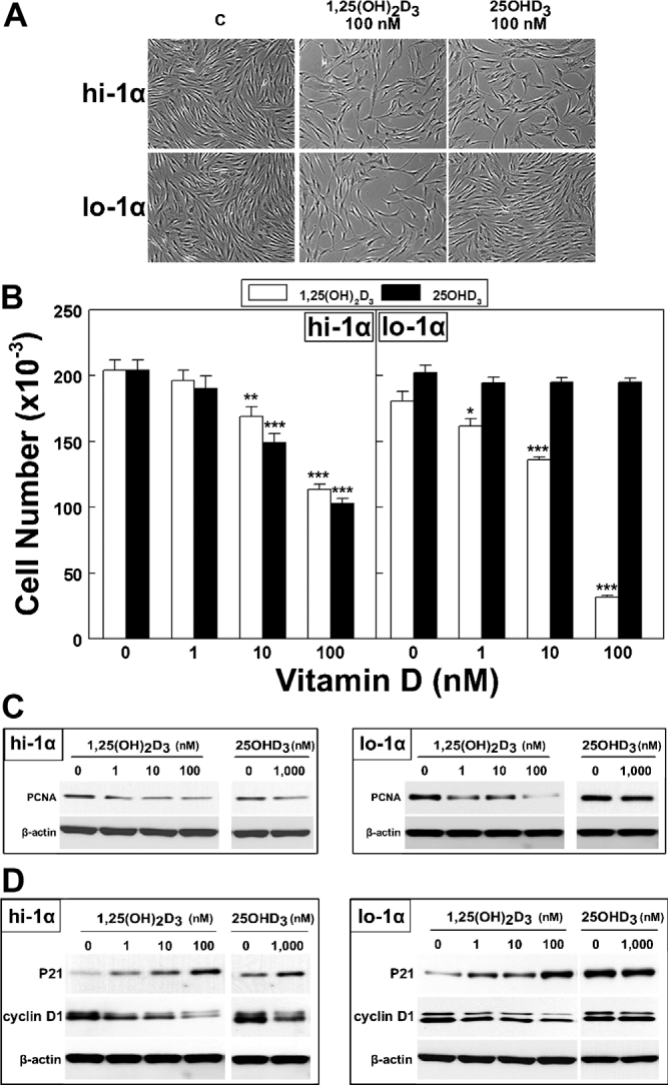
Relative effects of 25(OH)D_3_ and 1,25(OH)_2_D_3_ on proliferation of hMSCs. (*A*) Photomicrographs show hMSC^hi-1α^ and hMSC^lo-1α^ cultures after 3 days in the absence or presence of 100 nM 1,25(OH)_2_D_3_ or 25(OH)D_3_ (×200 magnification). (*B*) Cell number was determined in hMSC^hi-1α^ and hMSC^lo-1α^ cultures after 3 days in the absence or presence of 1, 10, 100 nM 1,25(OH)_2_D_3_ or 25(OH)D_3_. Results are expressed as mean ± SEM (12 replicate wells). *p < .05; **p < .01; ***p < .001. (*C*) Western immunoblots show proliferating cell nuclear antigen and β-actin levels in hMSC^hi-1α^ and hMSC^lo-1α^ cultures after 3 days in the absence or presence of 1,25(OH)_2_D_3_ or 25(OH)D_3_. (*D*) Western immunoblots show p21, cyclin D1, and β-actin in hMSC^hi-1α^ and hMSC^lo-1α^ cultures after 3 days in the absence or presence of 1,25(OH)_2_D_3_ or 25(OH)D_3_.

Two samples of hMSCs were cultured for 3 days after seeding in standard growth medium (10% FBS-HI). There was less cellularity in cultures of both hMSCs^hi-1α^ and hMSCs^lo-1α^ treated with 100 nM 1,25(OH)_2_D_3_ compared with vehicle control. In contrast, only the hMSCs^hi-1α^ were inhibited by 25(OH)D_3_ (Fig. 2*A*). There was a dose-dependent inhibition of proliferation with 1,25(OH)_2_D_3_ for both cell preparations (Fig. 2*B*). Both 25(OH)D_3_ and 1,25(OH)_2_D_3_ inhibited proliferation of hMSCs^hi-1α^; there was a significant inhibition of proliferation of hMSCs^hi-1α^ at 100 nM of 25(OH)D_3_ (56% of control cell number, *p* < .001) and 1,25(OH)_2_D_3_ (50%; *p* < .001). In contrast, hMSCs^lo-1α^ were resistant to 25(OH)D_3_ (96%) yet were inhibited by 1,25(OH)_2_D_3_ (17%, *p*< .001; Fig. 2*B*). Consistent with the effects on cell numbers, PCNA was downregulated by 1,25(OH)_2_D_3_ in hMSCs^hi-1α^ and in hMSCs^lo-1α^ (Fig. 2*C*). With 25(OH)D_3_ treatment, PCNA in hMSCs^hi-1α^ was 64.9% of control, but for hMSCs^lo-1α^, PCNA was equivalent to control. With hMSCs^hi-1α^, both 25(OH)D_3_ and 1,25(OH)_2_D_3_ downregulated cyclin D1 and upregulated the negative regulator p21^Waf1/Cip1^ (Fig. 2*D*). In hMSCs^lo-1α^, the effects of 1,25(OH)_2_D_3_ on cell cycle regulators were similar to those for hMSCs^hi-1α^, but there were no effects by 25(OH)D_3_.

### Relative effects of 25(OH)D_3_ and 1,25(OH)_2_D_3_ on Bax/Bcl-2 ratios in hMSCs

**Fig. 3:**
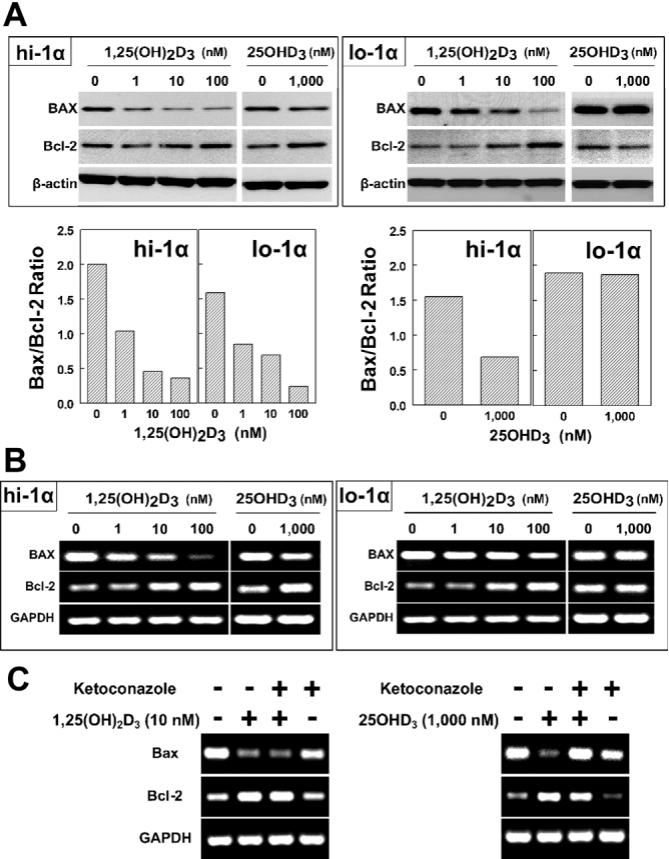
Relative effects of 25(OH)D_3_ and 1,25(OH)_2_D_3_ on Bax/Bcl-2 ratios in hMSCs. (*A*) Western immunoblots show Bax, Bcl-2, and β-actin in hMSC^hi-1α^ and hMSC^lo-1α^ cultures after 3 days in the absence or presence of 1,25(OH)_2_D_3_ or 25(OH)D_3_. The bar graphs represent the Bax/Bcl-2 ratios after each densitometric value was normalized to β-actin. (*B*) Gel electrophoretograms show RT-PCR products for *Bax*, *Bcl*-2, and *GAPDH* in hMSC^hi-1α^ and hMSC^lo-1α^ cultures after 3 days in the absence or presence of 1, 10, or 100 nM 1,25(OH)_2_D_3_ or 1000 nM 25(OH)D_3_. (*C*) Gel electrophoretogram shows RT-PCR products for *Bax*, *Bcl*-2, and *GAPDH* in hMSCs^hi-1α^ after 3 days in the absence or presence of 10 nM 1,25(OH)_2_D_3_ or 1000 nM 25(OH)D_3_ ± 10 µM ketoconazole.

Mechanisms involved in the relative effects of 25(OH)D_3_ and 1,25(OH)D_3_ were studied by analysis of expression of apoptosis-associated proteins. First, effects of metabolites were compared in hMSCs^hi-1α^ and hMSCs^lo-1α^. In hMSCs^hi-1α^, both 25(OH)D_3_ and 1,25(OH)_2_D_3_ induced a downregulation of Bax and an upregulation of the Bcl-2 protein (Fig. 3*A*); these effects resulted in lower Bax/Bcl-2 ratios with 100 nM 1,25(OH)_2_D_3_ (20% compared with vehicle control) and with 1000 nM 25(OH)D_3_ (43%). In hMSCs^lo-1α^, the Bax/Bcl-2 ratio was lower with 100 nM 1,25(OH)_2_D_3_ (19%), but there was essentially no effect by 25(OH)D_3_ (95%). The effects on mRNA levels of *Bax* and *Bcl*-2 (Fig. 3*B*) corresponded with the changes observed for protein levels (Fig. 3*A*).

As a second approach, the cytochrome P450 inhibitor ketoconazole was used to determine the importance of hydroxylation on 25(OH)D_3_ effects on proliferation. Ketoconazole (10 µM) diminished the effects of 25(OH)D_3_ (1000 nM) and not the effects of 1,25(OH)_2_D_3_ (10 nM) on Bax and Bcl-2 in hMSCs^hi-1α^ (Fig. 3*C*). In the presence of 25(OH)D_3_, the Bax/Bcl-2 ratio was 27% of that with vehicle control, and the decrease by 25(OH)D_3_ was blocked by ketoconazole (90%). In the presence of 1,25(OH)_2_D_3_, the Bax/Bcl-2 ratio was 36% of that with vehicle control, similar to that with 1,25(OH)_2_D_3_ and ketoconazole (37%).

### Relative effects of 25(OH)D_3_ and 1,25(OH)_2_D_3_ on osteoblast differentiation in hMSCs

**Fig. 4:**
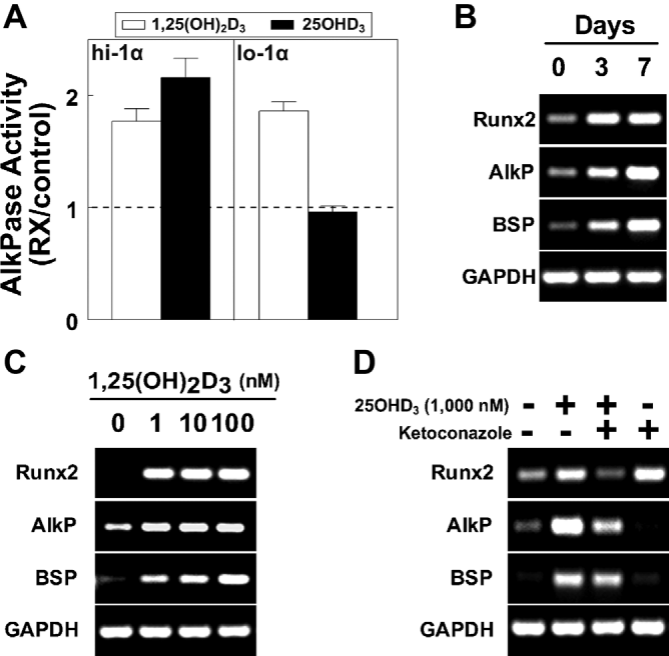
Comparison of effects of 25(OH)D_3_ and 1,25(OH)_2_D_3_ on osteoblast differentiation in hMSCs. (*A*) Alkaline phosphatase enzymatic activity (6 replicate wells) was measured in hMSCs^hi-1α^ and hMSCs^lo-1α^ in the absence or presence of 10 nM 1,25(OH)_2_D_3_ (*open bars*) or 25(OH)D_3_ (*closed bars*) in osteogenic medium with 1% FBS-HI for 7 days. Results are reported relative to control (Rx/control) with horizonal dashed line as 1.0; mean ± SEM. (*B*) Gel electrophoretogram shows RT-PCR products of osteoblast signature genes (*Runx2*, *AlkP*, and *BSP*) and *GAPDH* in hMSCs^hi-1α^ after 0, 3, and 7 days in standard osteogenic medium with 10% FBS-HI. (*C*) Gel electrophoretogram shows RT-PCR products of osteoblast signature genes (*Runx2*, *AlkP*, and *BSP*) and *GAPDH* in hMSCs^hi-1α^ after 3 days in the absence or presence of 1, 10, or 100 nM 1,25(OH)_2_D_3_ in standard growth medium with 10% FBS-HI. (*D*) Gel electrophoretogram shows RT-PCR products of osteoblast signature genes (*Runx2*, *AlkP*, and *BSP*) and *GAPDH* in hMSCs^hi-1α^ after 3 days in the absence or presence of 1000 nM 25(OH)D_3_ ± 10 µM ketoconazole in standard growth medium with 10% FBS-HI.

Regulation of osteoblast differentiation was quantified first by AlkP enzymatic activity assays in osteogenic medium with 1% FBS-HI (Fig. 4*A*). In hMSCs^hi-1α^, there was similar stimulation of AlkP activity by 25(OH)D_3_ (2.16-fold, *p* = .0003) and by 1,25(OH)_2_D_3_ (1.77-fold, *p* < .0001). In contrast, with hMSCs^lo-1α^, 25(OH)D_3_ had no effect (0.96-fold, *p* = .577), and 1,25(OH)_2_D_3_ stimulated AlkP activity (1.86-fold, *p* < .0001).

Osteoblast differentiation was also monitored by osteoblast signature genes (ie, *Runx2*, *AlkP*, and *BSP*) after transfer to standard osteogenic medium (10% FBS-HI) or after addition of 1,25(OH)_2_D_3_ to standard growth medium (10% FBS-HI). As expected, there was time-dependent upregulation of *Runx2*, *AlkP*, and *BSP* in hMSCs^hi-1α^ in standard osteogenic medium (Fig. 4*B*). Addition of 1,25(OH)_2_D_3_ to standard growth medium also upregulated osteoblast genes in hMSCs^hi-1α^ (Fig. 4*C*). Addition of 25(OH)D_3_ to standard growth medium also upregulated osteoblast genes in hMSCs^hi-1α^, but its effect was diminished by ketoconazole (Fig. 4*D*).

### Effect of *CYP27B1* siRNA on the stimulation of osteoblast differentiation by 25(OH)D_3_

**Fig. 5:**
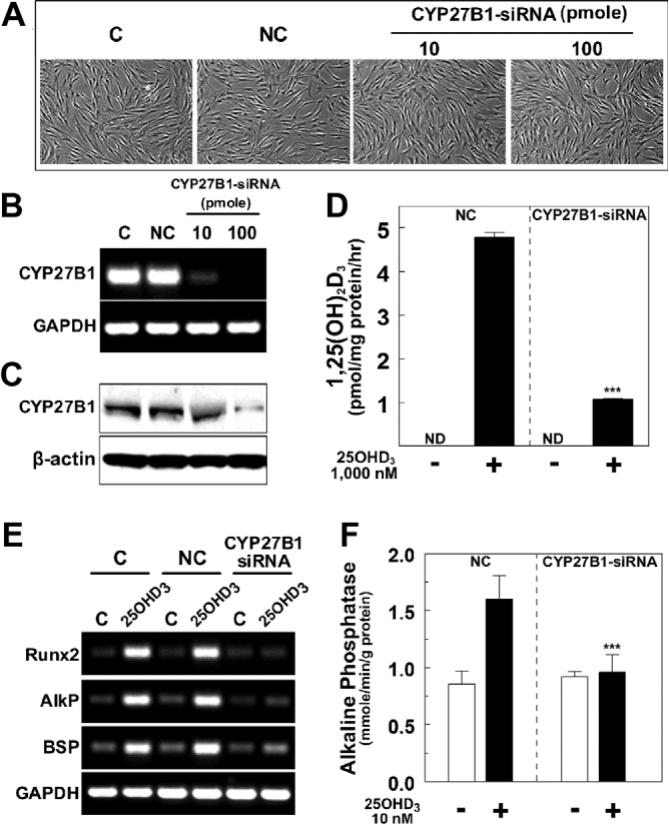
Effect of *CYP27B1* siRNA on the stimulation of osteoblast differentiation by 25(OH)D_3_. Four groups were treated by electroporation with PBS (C = control), with nonsilencing control siRNA (NC), or with 10 or 100 pmol of *CYP27B1* siRNA. (*A*) Photomicrographs show cultures of control and transfected hMSCs^hi-1α^ (×200 magnification). (*B*) Gel electrophoretogram shows *CYP27B1* and *GAPDH* in controls and in transfected cells. (*C*) Western immunoblot shows CYP27B1 and β-actin protein levels in controls and in transfected cells. (*D*) Cells transfected with nonsilencing siRNA and 100 pmol of *CYP27B1* siRNA were treated with or without 1000 nM 25(OH)D_3_ in serum-free α-MEM supplemented with 1% ITS^+1^, 10 µM 1,2-dianilinoethane (*N*,*N*'-diphenylethylene diamine) for 24 hours. Cellular 1,25(OH)_2_D_3_ production was determined by EIA as described under “In vitro biosynthesis of 1,25(OH)_2_D_3_ by hMSCs.” Results are shown as the mean ± SEM (3 replicate wells). There was no detectable (ND) 1,25(OH)_2_D_3_ in cultures without 1000 nM 25(OH)D_3_ exogenous substrate. ***p< .001. (*E*) Gel electrophoretogram shows *Runx2*, *AlkP*, *BSP*, and *GAPDH* in controls and in transfected hMSCs^hi-1α^ after 3 days ± 1000 nM 25(OH)D_3_. (*F*) Alkaline phosphatase enzymatic activity was measured in control and transfected hMSCs^hi-1α^ (100 pmol of *CYP27B1* siRNA) after 7 days ± 10 nM 25(OH)D_3_ in osteogenic medium. Values represent the mean ± SEM (6 replicate wells). ***p< .001.

As another approach to assess the mechanism by which 25(OH)D_3_ can stimulate osteoblast differentiation, hMSCs^hi-1α^ were engineered to have reduced constitutive expression of CYP27B1. There were no noticeable differences in cell density or appearance of control cells (electroporation with PBS), cells treated with nonsilencing control siRNA, and cells with 10 or 100 pmol *CYP27B1* siRNA (Fig. 5*A*). Transient transfection of *CYP27B1* siRNA into hMSCs^hi-1α^ resulted in reductions of *CYP27B1* mRNA (2% of control; Fig. 5*B*) and CYP27B1 protein (11% of control; Fig. 5*C*). No effect was shown with a nonsilencing, scrambled siRNA sequence (lane NC in Fig. 5*B, C*). The amount of 1,25(OH)_2_D_3_ synthesized by the cells transfected with *CYP27B1* siRNA was 22% of that for cells transfected with nonsilencing siRNA (1075 versus 4786 fmol/mg protein per hour, *p* < .0001; Fig. 5*D*). Treatment with 25(OH)D_3_ upregulated *Runx2*, *AlkP*, and *BSP* in both control preparations of hMSCs^hi-1α^. With cells transfected with *CYP27B1* siRNA, however, 25(OH)D_3_ had no effect on osteoblast genes (Fig. 5*E*). As a functional marker of osteoblast differentiation, we measured AlkP enzymatic activity after 7 days in osteogenic medium (1% FBS-HI). Whereas 25(OH)D_3_ stimulated AlkP activity of control cells (1.87-fold, *p* < .0001), there was no effect in cells transfected with *CYP27B1* siRNA (1.04-fold, *p* = .093; Fig. 5*F*).

## Discussion

This study used three approaches to examine the role of CYP27B1 on the effects of 25(OH)D_3_ in hMSCs. First, we compared cells with high and low constitutive expression of CYP27B1. Finding a wide range of expression in hMSCs from 22 subjects is consistent with our previous studies.(17) The level of CYP27B1 expression was found to be related to the vitamin D status(17) and, more recently, to age(25) of the subjects. There is growing evidence that hMSCs(17) and human bone cells(26) are both sources and targets of 1,25(OH)_2_D_3_, and thus vitamin D may have multiple autocrine/paracrine actions in bones.

It was important to control for 24-hydroxylation in these studies because differences in inactivation of added vitamin D metabolites could confound interpretation. The activities of CYP27B1 and CYP24A1 are important for the maintenance of appropriate levels of 1,25(OH)_2_D_3_ and 25(OH)D_3_. Therefore, two hMSCs were selected and studied in detail on the basis of having extremes in expression of CYP27B1 and equivalent expression of CYP24A1 and VDR. Further, the expression of CYP24A1 was found to be regulated with equivalence in both specimens of hMSCs; this observation reduces concerns of confounding effects of 24-hydroxylation in these studies. In addition, fresh metabolites were added daily.

There were substantial differences in the synthesis of 1,25(OH)_2_D_3_ by hMSCs^hi-1α^ and hMSCs^lo-1α^. The difference also held for hMSCs with and without *CYP27B1* gene silencing. Although these experiments cannot be used to estimate what would be the steady-state concentration of 1,25(OH)_2_D_3_ in the bone marrow in different subjects whose cells have high or low expression of CYP27B1, they provide evidence for a potential autocrine/paracrine role for 25(OH)D_3_ metabolism in osteoblast differentiation. Similar ideas have been proposed for 25(OH)D_3_ metabolism in regulating bone matrix formation by differentiated human osteoblasts.(23)

There was dose-dependent inhibition of proliferation by 25(OH)D_3_ with hMSCs that had a high level of expression of CYP27B1; 25(OH)D_3_ reduced their proliferation and downregulated PCNA. There is some information about antiproliferative actions of 25(OH)D_3_ in other human cell types. In human primary prostate epithelial cells that expressed CYP27B1, low concentrations of 25(OH)D_3_ suppressed cell growth.(27) In prostatic cancer cells lacking CYP27B1, 25(OH)D_3_ failed to demonstrate antiproliferative action.(28) There are several mechanisms mediating the antiproliferative effects of 1,25(OH)_2_D_3_. In U937 myelomonocytic cells, 1,25(OH)_2_D_3_ induces an arrest in the G_1_ phase of the cell cycle that depends on upregulation of the cyclin-dependent kinase inhibitor p21^Waf1/Cip1^.(29) More recently, p21^Waf1/Cip1^ was shown to be a primary antiproliferative mediator for the VDR in the presence of its ligand, 1,25(OH)_2_D_3_.(30) Cyclin D1 is increased in dividing cells during the G_1_ phase and is necessary for the transition from G_1_ to S phase.(31) Vitamin D decreases cyclin D1 abundance and/or activity by different mechanisms in different cell types. For example, in human epidermoid A431 cells, 1,25(OH)_2_D_3_ inhibited transforming growth factor α (TGF-α)/endothelial growth factor receptor (EGFR) transactivation of cyclin D1.(32) We found that 25(OH)D_3_ downregulated cyclin D1 and upregulated the negative regulator p21^Waf1/Cip1^ in hMSCs^hi-1α^. In contrast, 25(OH)D_3_ had no such effects in hMSCs^lo-1α^. The upregulation of p21^Waf1/Cip1^ and decreased expression of cyclin D1 in hMSCs^hi-1α^ provide mechanisms for the antiproliferative effect of 25(OH)D_3_.

1,25(OH)_2_D_3_ also affects the levels of proapoptotic (ie, Bax and Bak) and antiapoptotic (ie, Bcl-2 and Bcl-XL) proteins, resulting in apoptosis in several tumor models, including human carcinomas of the breast, colon, and prostate.(4,10,33) This study with hMSCs indicates that the antiproliferation effects of 1,25(OH)_2_D_3_ or 25(OH)D_3_ are not explained by increases in Bax or decreases in Bcl-2. In fact, the ratio of Bax/Bcl-2 decreases at both the mRNA and protein levels. Two lines of evidence indicate that those effects of 25(OH)D_3_ on Bax and Bcl-2 depend on CYP27B1. First, they were not detected in hMSCs^lo-1α^. Second, they were blocked in hMSCs^hi-1α^ by the pan-cytochrome P450 inhibitor ketoconazole, not like the effects of 1,25(OH)_2_D_3_, which were not affected by ketoconazole. The antiapoptotic effects of 1,25(OH)_2_D_3_ or 25(OH)D_3_ in hMSCs^hi-1α^ are different from the proapoptotic effects in some cancer cells(4,10,33) and are similar to the effects in other cell types. In ovarian cancer cells, 1,25(OH)_2_D_3_ inhibits apoptosis that is mediated by death receptors.(34) In rat osteoblast-like osteosarcoma UMR 106 cells, 1,25(OH)_2_D_3_ elicited antiapoptotic effects by decreasing the Bax/Bcl-2 ratio.(11) There are other antiapoptotic signals, as was reported for nongenotropic mechanisms in osteoblasts and osteocytes.(35) In sum, the data indicate that the antiproliferative effects of 25(OH)D_3_ in hMSCs^hi-1α^ and of 1,25(OH)_2_D_3_ in both samples of hMSCs are explained by cell cycle arrest and not by increased apoptosis.

Calcitriol induces differentiation of many types of benign and malignant cells.(12,13,18,36–38) The differentiation of various cells, including human myelomonocytic cells,(9,29) induced by 1,25(OH)_2_D_3_ depends on the induction of p21^Waf1/Cip1^. Further, 1,25(OH)_2_D_3_ is a key regulator of the reciprocal relationship between proliferation and differentiation during the osteoblast development sequence.(39) Vitamin D or its analogues promote osteoblastic differentiation, as shown for osteosarcoma cell lines MG-63,(40) HOS,(23) SAOS,(41) and TE85.(41) Osteoblast differentiation of human MSCs is stimulated by 1,25(OH)_2_D_3_ or 25(OH)D_3_ in hMSCs^hi-1α^, as shown here and elsewhere.(17,18,37,38,42) As expected, 25(OH)D_3_ failed to stimulate osteoblast differentiation in hMSCs^lo-1α^. Upregulation of osteoblast genes by 25(OH)D_3_ in hMSCs^hi-1α^ was diminished by ketoconazole. Thus experiments with ketoconazole indicate that both the antiproliferative and prodifferentiation effects of 25(OH)D_3_ depend on CYP27B1.

Ketoconazole is a strong but differential inhibitor of both CYP24A1 and CYP27B1(43) and may be cytotoxic for some cells.(23) Those confounders may complicate interpretation of results obtained with this agent. We therefore used the highly specific technique of RNA interference to inhibit CYP27B1 expression in hMSCs^hi-1α^. The level of synthesized 1,25(OH)_2_D_3_ in the cells transfected with *CYP27B1* siRNA was reduced to 22% of that for cells transfected with nonsilencing siRNA. Osteoblast differentiation of hMSCs^hi-1α^ by 25(OH)D_3_ was prevented upon transfection with *CYP27B1* siRNA, as indicated by osteoblast signature gene expression and by AlkP enzymatic activity. These findings are consistent with those from a study with HOS human osteosarcoma cells in which silencing of *CYP27B1* resulted in a suppression of 25(OH)D_3_'s effects on those cells.(23,44)

In conclusion, 25(OH)D_3_ has multiple effects in normal hMSCs; it inhibits proliferation and promotes osteoblast differentiation by mechanisms similar to those for 1,25(OH)_2_D_3_. Our data indicate that antiproliferative and prodifferentiation effects of 25(OH)D_3_ in hMSCs require 1α-hydroxylase. There are suggestions that other effects of 25(OH)D_3_ in other cell types, such as induction of 24-hydroxylase in prostatic cells, may not require 1α-hydroxylase.(45) Three lines of evidence indicate that CYP27B1 is required for the effects of 25(OH)D_3_ on hMSCs. Those effects were not seen (1) in hMSCs with low constitutive expression of CYP27B1, (2) in hMSCs treated with ketoconazole, or (3) in hMSCs in which CYP27B1 expression was silenced. These findings suggest that local osteoblast differentiation in vivo may be promoted by 25(OH)D_3_ if the progenitor/precursor cells in marrow express CYP27B1/1α-hydroxylase. We found that many of hMSCs' in vitro behaviors and baseline characteristics depend on clinical features of the subjects from whom the cells were isolated. The level of CYP27B1 expression in those cells depends on vitamin D status and can be regulated by a number of factors, including vitamin D.(17) The combined presence of CYP27B1 and the VDR in hMSCs indicates possible autocrine/paracrine roles for 25(OH)D_3_ to regulate osteoblast differentiation and skeletal homeostasis.
